# Forecasting stock prices with long-short term memory neural network based on attention mechanism

**DOI:** 10.1371/journal.pone.0227222

**Published:** 2020-01-03

**Authors:** Jiayu Qiu, Bin Wang, Changjun Zhou

**Affiliations:** 1 Key Laboratory of Advanced Design and Intelligent Computing (Dalian University), Ministry of Education, Dalian, China; 2 College of Computer Science and Engineering, Dalian Minzu University, Dalian, China; Polytechnical Universidad de Madrid, SPAIN

## Abstract

The stock market is known for its extreme complexity and volatility, and people are always looking for an accurate and effective way to guide stock trading. Long short-term memory (LSTM) neural networks are developed by recurrent neural networks (RNN) and have significant application value in many fields. In addition, LSTM avoids long-term dependence issues due to its unique storage unit structure, and it helps predict financial time series. Based on LSTM and an attention mechanism, a wavelet transform is used to denoise historical stock data, extract and train its features, and establish the prediction model of a stock price. We compared the results with the other three models, including the LSTM model, the LSTM model with wavelet denoising and the gated recurrent unit(GRU) neural network model on S&P 500, DJIA, HSI datasets. Results from experiments on the S&P 500 and DJIA datasets show that the coefficient of determination of the attention-based LSTM model is both higher than 0.94, and the mean square error of our model is both lower than 0.05.

## Introduction

Financial market forecasting has traditionally been a focus of industry and academia.[1] For the stock market, its volatility is complicated and nonlinear.[2] It is obviously unreliable and inefficient to rely solely on a trader’s personal experience and intuition for analysis and judgment. People need an intelligent, scientific, and effective research method to direct stock trading. With the rapid development of artificial intelligence, the application of deep learning in predicting stock prices has become a research hotspot. The neural network in deep learning has become a popular predictor due to its good nonlinear approximation ability and adaptive self-learning. Long short-term memory (LSTM) neural networks have performed well in speech recognition[3, 4] and text processing.[5, 6] At the same time, because they have the characteristics of selectivity, memory cells, LSTM neural networks are suitable for random nonstationary sequences such as stock-price time series.

Due to the nonstationary, nonlinear, high-noise characteristics of financial time series,[7] traditional statistical models have difficulty predicting them with high precision. Although there are still some difficulties and problems in financial predictions using deep learning, people hope to establish a reliable stock market forecasting model.[8] Increased attempts are being made to apply deep learning to stock market forecasts. In 2013, Lin et al.[9] proposed a method to predict stocks using a support vector machine to establish a two-part feature selection and prediction model and proved that the method has better generalization than conventional methods. In 2014, Wanjawa et al. [10] proposed an artificial neural network using a feed-forward multilayer perceptron with error backpropagation to predict stock prices. The results show that the model can predict a typical stock market. Later, Zhang et al.[11] combined convolutional neural network (CNN) and recurrent neural network (RNN) to propose a new architecture, the deep and wide area neural network (DWNN). The results show that the DWNN model can reduce the predicted mean square error by 30% compared to the general RNN model. There have been many recent studies on the application of LSTM neural networks to the stock market. A hybrid model of generalized autoregressive conditional heteroskedasticity (GARCH) combined with LSTM was proposed to predict stock price fluctuations.[12] CNN was used to develop a quantitative stock selection strategy to determine stock trends and then predict stock prices using LSTM to promote a hybrid neural network model for quantitative timing strategies to increase profits.[13] A time-weighted function was added to an LSTM neural network, and the results surpassed those of other models.[14] Jiang et al.[15] used an LSTM neural network and RNN to construct models and found that LSTM could be better applied to stock forecasting. Jin et al.[16] added investor sentiment tendency in model analysis and introduced empirical modal decomposition (EMD) combined with LSTM to obtain more accurate stock forecasts. The LSTM model based on the attention mechanism is common in speech and image recognition but is rarely used in finance.

## Related theory and technology

### Long short-term memory neural networks (LSTM)

**Fig 1:**
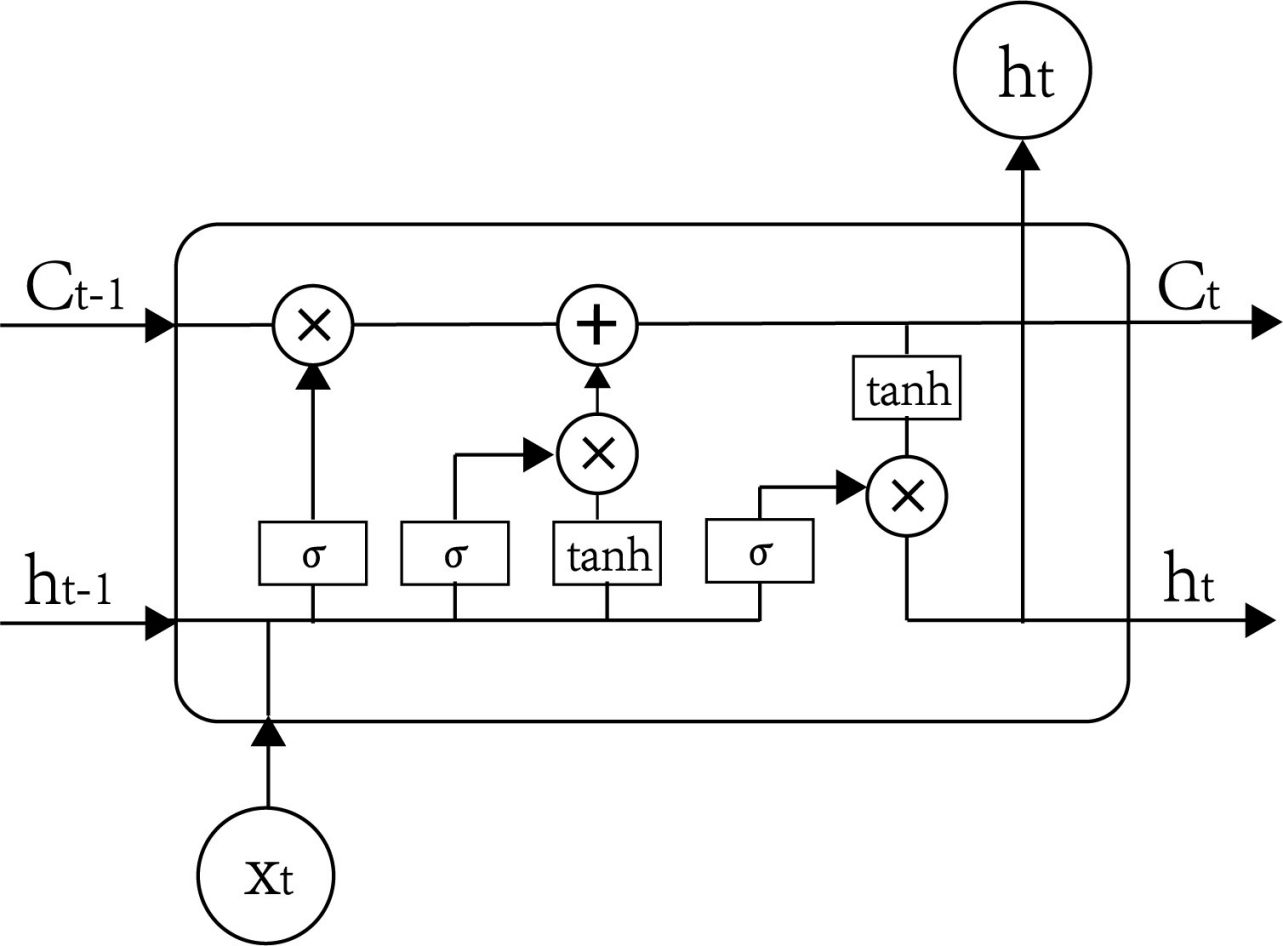
Structure of long short term memory(LSTM).

LSTM uses one of the most common forms of RNN.[17] This time recurrent neural network is meant to avoid long-term dependence problems and is suitable for processing and predicting time series. Proposed by Sepp Hochreiter and Jurgen Schmidhuber in 1997,[18] the LSTM model consists of a unique set of memory cells that replace the hidden layer neurons of the RNN, and its key is the state of the memory cells. The LSTM model filters information through the gate structure to maintain and update the state of memory cells. Its door structure includes input, forgotten, and output gates. Each memory cell has three sigmoid layers and one tanh layer. Fig 1 displays the structure of LSTM memory cells.

The forgotten gate in the LSTM unit determines which cell state information is discarded from the model. As shown in Fig 1, the memory cell accepts the output *h*_*t-1*_ of the previous moment and the external information *x*_*t*_ of the current moment as inputs and combines them in a long vector [*h*_*t-1*_, *x*_*t*_] through σ transformation to become

$$f_{t}=\sigma (W_{f}∙[h_{t-1},x_{t}]+b_{f}),$$ (1)

where *W*_*f*_ and *b*_*f*_ are, respectively, the weight matrix and bias of the forgotten gate, and *σ* is the sigmoid function. The forgotten gate’s main function is to record how much the cell state *C*_*t-1*_ of the previous time is reserved to the cell state *C*_*t*_ of the current time. The gate will output a value between 0 and 1 based on *h*_*t-1*_ and *x*_*t*_, where 1 indicates complete reservation and 0 indicates complete discardment.

The input gate determines how much of the current time network input *x*_*t*_ is reserved into the cell state *C*_*t*_, which prevents insignificant content from entering the memory cells. It has two functions. One is to find the state of the cell that must be updated; the value to be updated is selected by the sigmoid layer, as in Eq (2). The other is to update the information to be updated to the cell state. A new candidate vector ${\hat{C}}_{t}$ is created through the tanh layer to control how much new information is added, as in Eq (3). Finally, Eq (4) is used to update the cell state of the memory cells:

$$i_{t}=\sigma (W_{t}∙[h_{t-1},x_{t}]+b_{i}),$$ (2)

$${\hat{C}}_{t}=tanh(W_{c}∙[h_{t-1},x_{t}]+b_{c}),$$ (3)

$$C_{t}=f_{t}*C_{t-1}+i_{t}*{\hat{C}}_{t}.$$ (4)

The output gate controls how much of the current cell state is discarded. The output information is first determined by a sigmoid layer, and then the cell state is processed by tanh and multiplied by the output of the sigmoid layer to obtain the final output portion:

$$O_{t}=\sigma (W_{\sigma }∙[h_{t-1},x_{t}]+b_{o}).$$ (5)

The final output value of the cell is defined as:

$$h_{t}=O_{t}*tanh(C_{t}).$$ (6)

### Attention mechanism

Many algorithms and mechanisms are inspired by biological phenomena. For example, inspired by the astrocytes in the biological nervous system that can greatly regulate the operation of neurons, Song et al.[19–21] proposed a spiking neural P system with gel-like control functions. Similarly, derived from the study of human vision, the attention mechanism highlights important local information by allocating adequate attention to key information. The attention mechanism is excellent in serialized data such as speech recognition, machine translation, and part-of-speech tagging. Neural networks based on attention mechanisms have attracted great interest in deep learning research. Mnih et al.[22] used the attention mechanism on the RNN model to classify images, focusing on an image’s essential parts to reduce the task’s complexity. Bahdanau et al.[23] applied the attention mechanism to the natural language processing (NLP) field for the first time. They applied it to machine translation to enable simultaneous translation and alignment.

The attention mechanism is applied in stock forecasting mainly through the extraction of information in the news in an auxiliary role to judge price fluctuations. For example, Liu[24] proposed an attention-based cyclic neural network to train financial news to predict stock prices. Attention mechanisms can have either soft or hard attention. The hard attention mechanism focuses on one element in the input information, selecting information based on either maximum or random sampling, which requires much training to obtain good results. The soft attention mechanism assigns weight to all input information, enables more efficient use of input information, and obtains results in a timely manner. The soft attention mechanism can be formulated as

$$e_{t}=tanh(w_{a}[x_{1},x_{2},\ldots ,x_{T}]+b)$$ (7)

$$a_{t}=\frac{exp(e_{t})}{\sum_{k=1}^{T}exp(e_{k})},$$ (8)

where *w*_*a*_ is the weight matrix of the attention mechanism, indicating information that should be emphasized; *e*_*t*_ is the result of the first weighting calculation; *b* is the deviation of the attention mechanism; [*x*_*1*_, *x*_*2*_,*…*, *x*_*T*_] is the input of the attention mechanism, i.e., the output of the LSTM hidden layer; and *a*_*t*_ is the final weight obtained by [*x*_*1*_, *x*_*2*_,*…*, *x*_*T*_].

**Fig 2:**
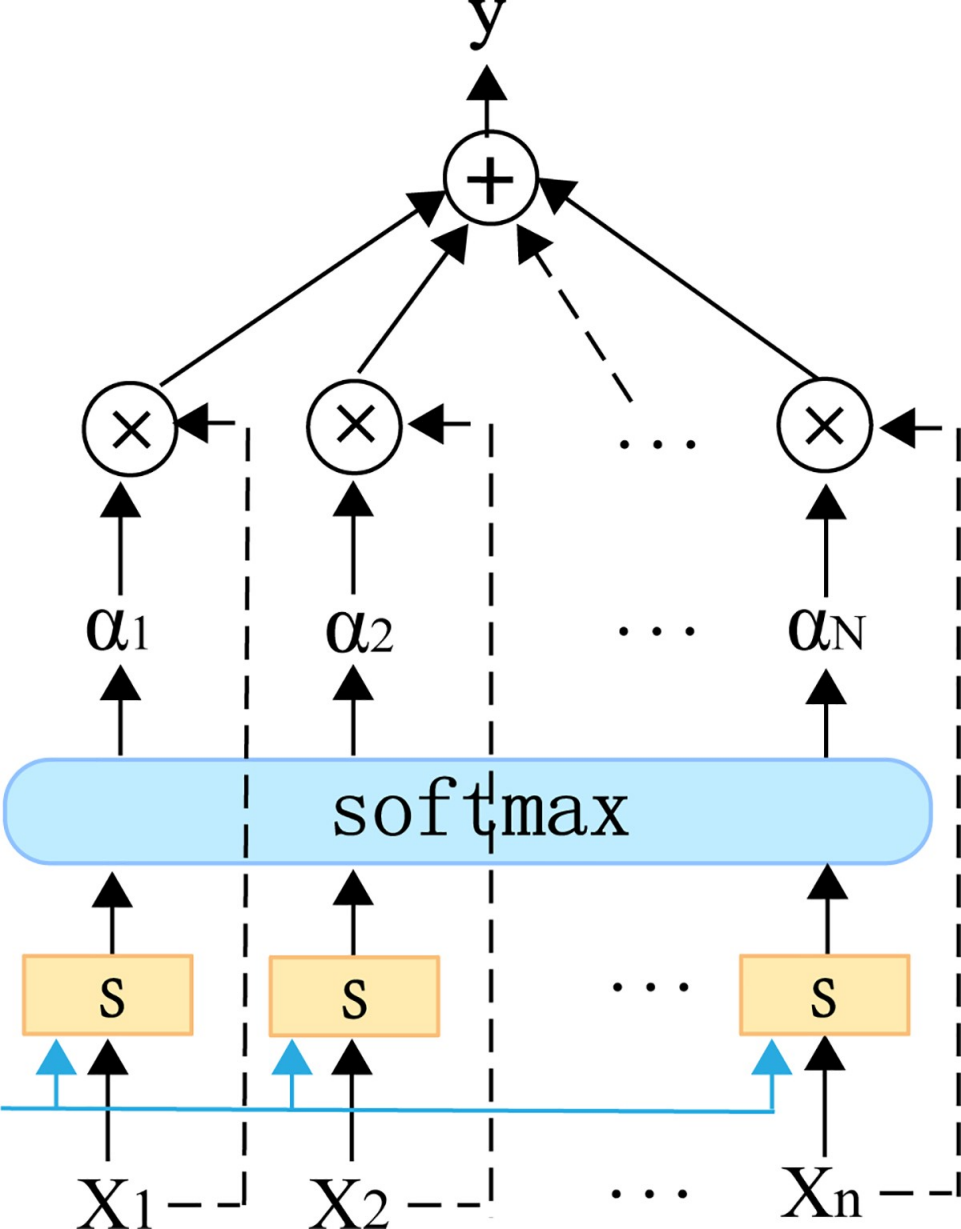
Basic structure of the attention model.

We calculate the degree of matching of each element in the input information and then input the matching degree score to a softmax function to generate the attention distribution. The general attention mechanism has two steps: (1) calculate the attention distribution; and (2) calculate the weighted average of the input information according to the attention distribution. We use x = [*x*_*1*_, *x*_*2*_,*…*, *x*_*N*_] to represent the N items of input information, pass it to the attention scoring function s, and input the result to the softmax layer to obtain the attention weight [*α*_*1*_, *α*_*2*_,*…*, *α*_*N*_]. Finally, the attention weight vector is weighted and averaged with the input information to obtain the final result. The attention value is obtained as shown in Fig 2.

### Wavelet transform

Wavelet analysis has led to remarkable achievements in areas such as image and signal processing.[25–27] With its ability to compensate for the shortcomings of Fourier analysis, it has gradually been introduced in the economic and financial fields. The wavelet transform has unique advantages in solving traditional time series analysis problems. It can decompose and reconstruct financial time series data from different time and frequency domain scales. Therefore, combining wavelet analysis theory with traditional time series theory enables us to better analyze and resolve problems in financial time series.

Financial time series have some of the same characteristics as signals analyzed in engineering. Therefore, a financial time series can be considered a signal. Wavelet threshold denoising has the basic idea to wavelet transform a signal, where the wavelet coefficient of the noise generated by wavelet decomposition is smaller than that of the signal. A threshold is selected to separate the useful signal from the noise, and the noise is then set to zero. The basic steps are wavelet decomposition, threshold processing, and reconstruction of signals. To realize this method depends on four factors: (1) selection of a wavelet basis function; (2) determination of the number of decomposition layers; (3) determination of the threshold value; and (4) selection of the threshold function. Commonly used wavelet basis functions are the Haar, db N, sym N, coif N, Morlet, Daubechies, and spline wavelet, among which the first four are relatively suitable for financial data denoising.

## Proposed prediction model

**Fig 3:**
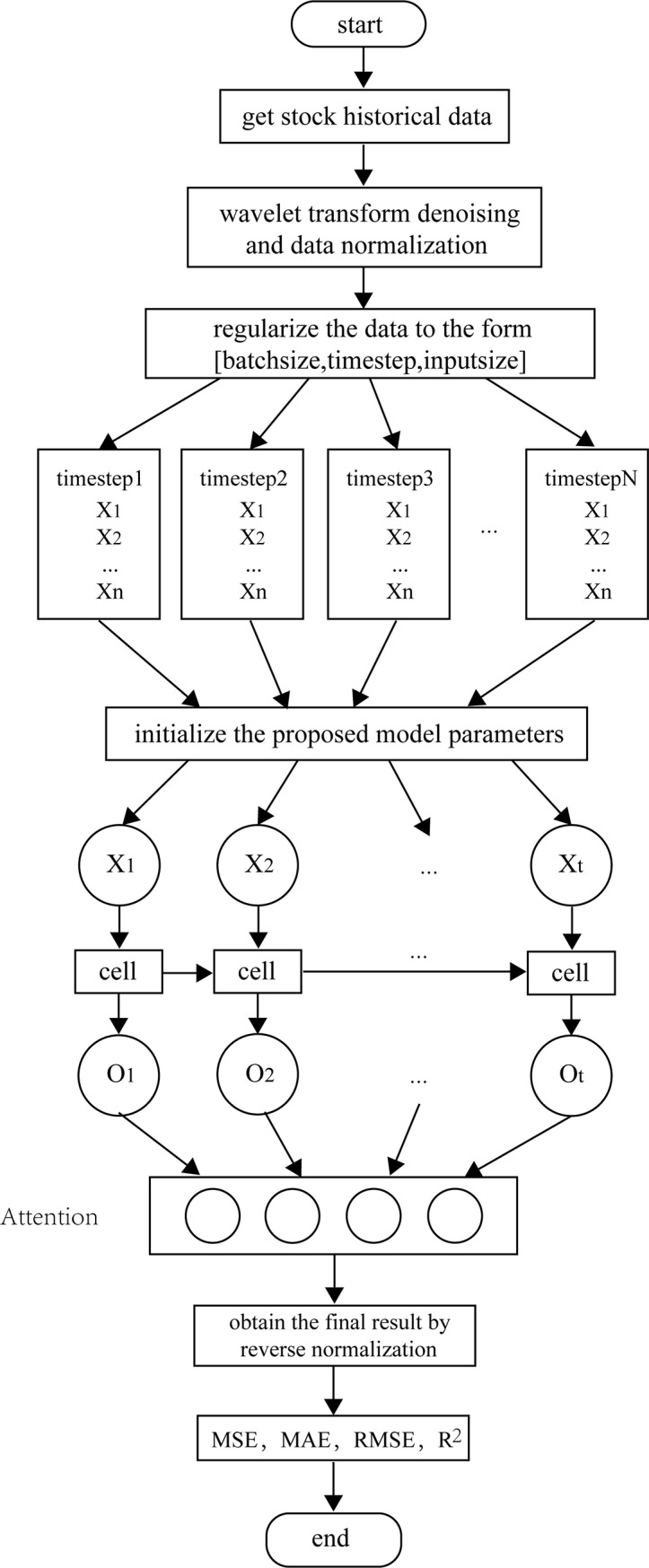
Attention-based LSTM model flowchart.

**Fig 4:**
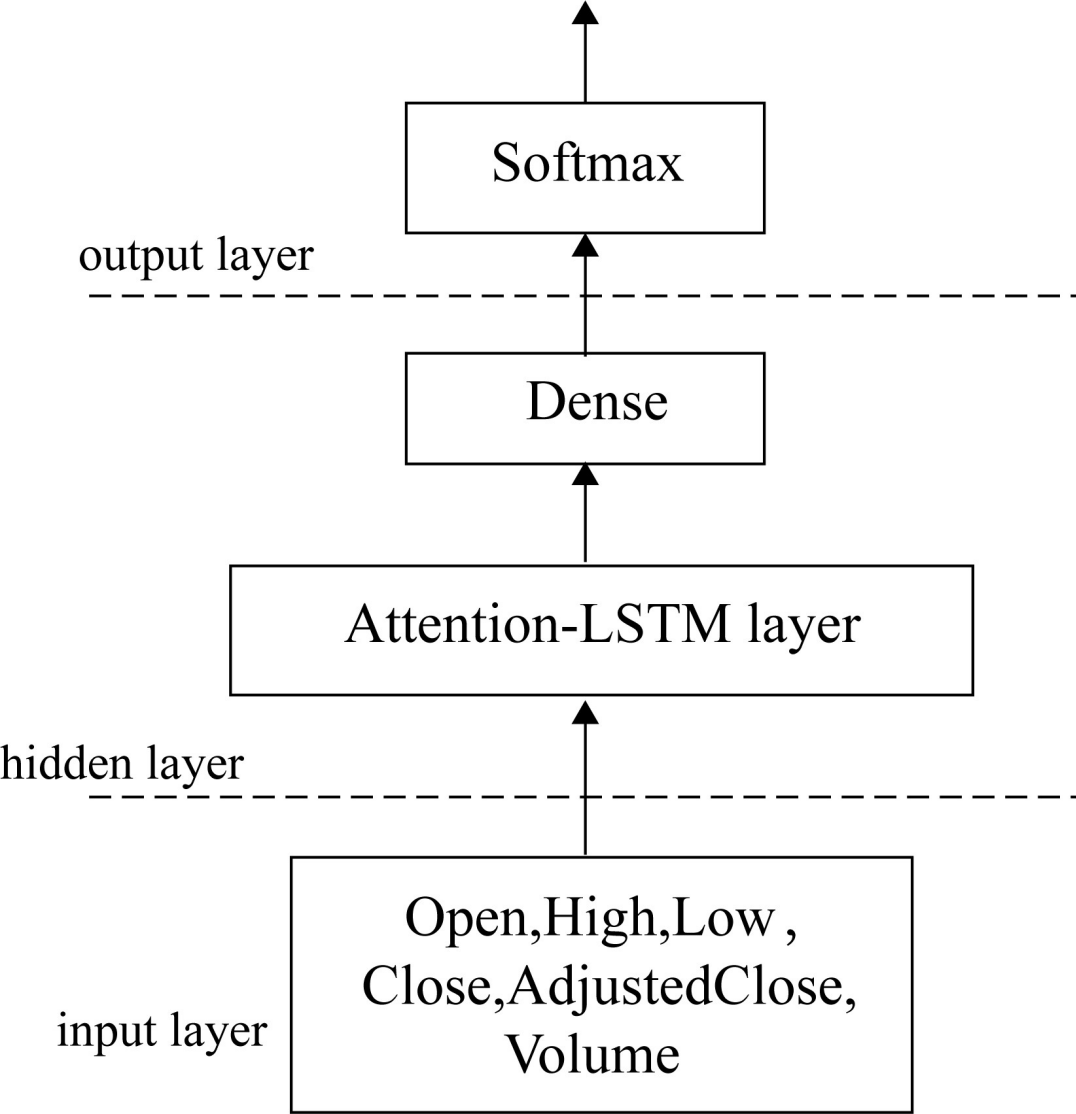
LSTM-Attention network structure.

To establish a stock index price forecasting model has three stages: data collection and preprocessing, model establishment and training, and evaluation of experimental results, as shown in Fig 3. As shown in Fig 4, the LSTM-Attention network structure consists of data input, hidden, and output layers, and the hidden layer consists of an LSTM, attention, and dense layer.

### Data source

We selected three stock indices as experimental data: the S&P 500 Index (S&P 500), Dow Jones Industrial Average (DJIA), and Hang Seng index (HSI). The S&P 500 and DJIA data are from January 3, 2000, to July 1, 2019. The HSI data are from January 2, 2002, to July 1, 2019. These data are from Yahoo! Finance. There are six variables in the basic transaction dataset. The opening price (open) is the first transaction price per share of a security after the market opens on a trading day, and the closing price (close) is its final price that day. High is the highest price a stock trades in a day, and low is the lowest price that day. Adjusted close is the closing price after adjustments for splits and dividend distributions. Data are adjusted using appropriate split and dividend multipliers. Volume refers to the number of transactions in a time unit for a transaction. We have listed some data samples of the S&P 500 in Table 1.

**Table 1:** Partial Stock Data Samples for S&P 500.

Date	Open	High	Low	Close	Adj Close	Volume
2000-01-03	1469.250000	1478.000000	1438.359985	1455.219971	1455.219971	931800000
2000-01-04	1455.219971	1455.219971	1397.430054	1399.420044	1399.420044	1009000000
…	…	…	…	…	…	…
2019-06-25	2945.780029	2946.520020	2916.010010	2917.379883	2917.379883	3578050000
2019-06-26	2926.070068	2932.590088	2912.989990	2913.780029	2913.780029	3478130000

### Data preprocessing

We implemented the proposed stock forecasting method in Python using TensorFlow. We used zero-mean normalization to the data and divided it into training and test datasets. For the S&P 500 and DJIA datasets, data from January 3, 2000, to May 16, 2019, were used for model training, and data from May 17, 2019, to July 1, 2019, were used for testing. For the HSI dataset, data from January 2, 2002, to May 16, 2019, were used for training, and from May 17, 2019, to July 1, 2019, for testing.

Due to the complex and volatile stock market and various trading restrictions, the stock prices we see are noisy.[28] At the same time, the financial time series is nonstationary and exhibits the overlapping of useful signals and noise, which makes traditional denoising ineffective. The wavelet transform is considered more suitable for extremely irregular financial sequences because it can perform both time domain and frequency domain analysis.[29] It combines with the traditional theory of time series analysis and shows good applicability. Therefore, wavelet analysis has become a powerful tool to process financial time series data.[30] We use a wavelet transform with multi-scale characteristics to denoise the dataset, effectively separating the useful signal from the noise. Specifically, we use the coif3 wavelet function with three decomposition layers, and we evaluate the effect of the wavelet transform by its signal-to-noise ratio (SNR) and root mean square error (RMSE). The higher the SNR and the smaller the RMSE, the better the denoising effect of the wavelet transform:

$$\mathrm{SNR}=10\log[\frac{\sum_{j=1}^{N}x_{j}{}^{2}}{\sum_{j=1}^{N}{\left(x_{j}-\hat{x_{j}}\right)}^{2}}].$$ (9)

### LSTM-Attention model

Because the input data include two types of data, i.e., stock price and volume, we use standardization and normalization to process the data to improve the training effect of the neural network model. Finally, the denoised data are input to the LSTM-Attention training model. The data are normalized to the form [*B*, *T*, *D*], where *B* is the batch size, *T* is the time step, and *D* is the dimension of the input data. The data are presented in the following form: $\left[\begin{array}{ccc} x_{1}^{\left(1\right)} & \cdots & x_{1}^{\left(D\right)}\\ \vdots & \ddots & \vdots \\ x_{T}^{\left(1\right)} & \cdots & x_{T}^{\left(D\right)} \end{array}\right]$. Each matrix is used as input data for a time step. The parameters of the model are initialized, and the processed input data are sequentially transmitted to the cells in the LSTM layer. Take the output value from the previous cell and use it as input to the attention layer. The proposed model network structure is an LSTM cyclic network with 10 hidden nodes per layer. The learning rate is set to 0.0001, and the number of iterations is 600.

## Experiment

### Model performance metrics

We evaluated the prediction results and the established prediction model by the mean square error (MSE), root mean square error (RMSE), mean absolute error (MAE), and coefficient of determination (R^2^). The smaller the MSE, RMSE, and MAE, the closer the predicted value to the true value; the closer the coefficient R^2^ to 1, the better the fit of the model:

$$MSE=\frac{1}{N}\sum_{i=1}^{N}\left(\hat{y_{i}}-y_{i}\right),$$ (10)

$$MAE=\frac{1}{N}\sum_{i=1}^{N}\left|\left(\hat{y_{i}}-y_{i}\right)\right|,$$ (11)

$$RMSE=\sqrt{\frac{1}{N}\sum_{i=1}^{N}\left(\hat{y_{i}}-y_{i}\right)},$$ (12)

$$R^{2}=\sum_{i=1}^{N}{\left(\hat{y_{i}}-\bar{y}\right)}^{2}/\sum_{i=1}^{N}{\left(y_{i}-\bar{y}\right)}^{2},$$ (13)

where N denotes the number of samples, $\hat{y_{i}}$ is the model prediction value, y_i_ is the real value, and $\bar{y}$ is the mean value of y_i_.

### Experimental results and discussion

**Fig 5:**
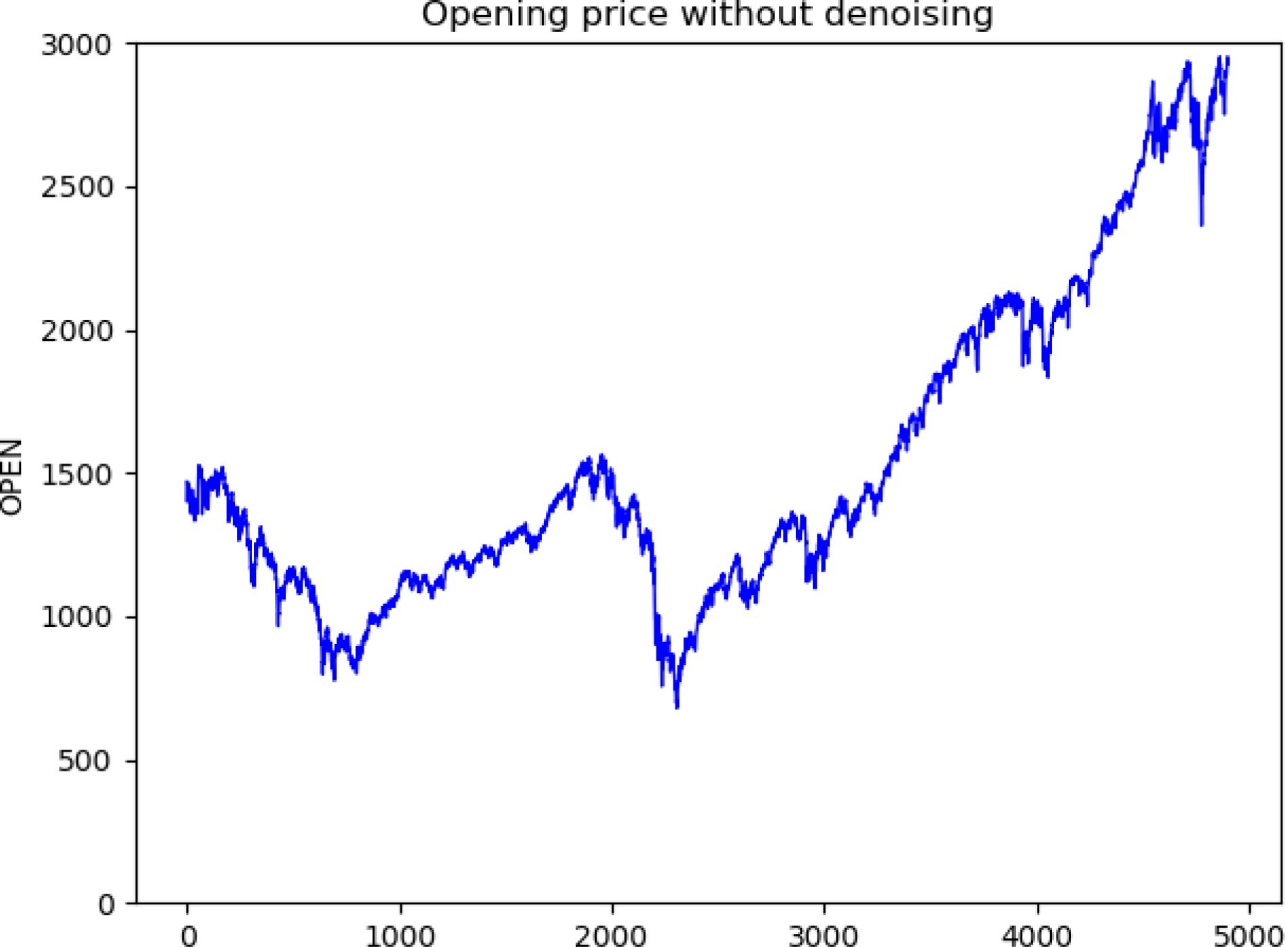
Opening price without denoising.

**Fig 6:**
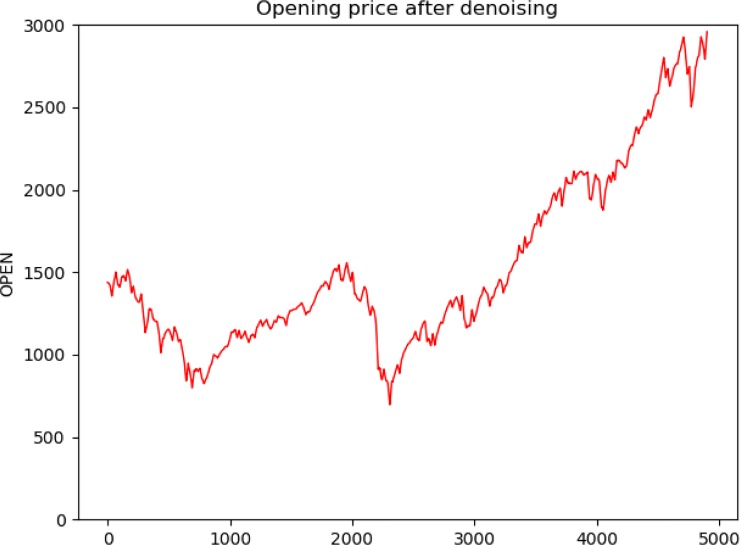
Opening price with denoising.

We experimented with different wavelet functions and used SNR and RMSE values to determine which wavelet was more suitable for stock price denoising. From the results in Table 2, we found that the SNR values of coif3 were the largest and the RMSE values were the smallest among the four wavelet functions. Therefore, we chose coif3 as the wavelet function for the experiment. Fig 5 shows the opening price curve before denoising using the wavelet transform. Fig 6 shows the opening price curve after denoising using the wavelet function. By comparing the two, it is found that the noise after wavelet transform processing is smaller.

**Table 2:** Comparison of evaluation indices of wavelet function denoising results.

	Sym3	Db3	Coif3	Haar
**SNR**	93.93	93.93	**95.03**	90.27
**RMSE**	14.78	14.78	**13.99**	17.75

We processed three stock index datasets in the LSTM model: the LSTM (WLSTM) model with the wavelet transform, the gated recurrent unit (GRU) neural network model, and our proposed WLSTM+Attention model. We trained them and compared the predicted results. Tables 3−5 show the evaluation indicators of the four prediction models on the S&P 500, DJIA, and HSI datasets, respectively. The results show that on the S&P 500 and DJIA datasets, our proposed model is significantly better than the other models, with an R^2^ average of 0.95. However, on the HSI dataset, although the proposed model is superior to the others, the error and model fit are significantly worse than on the other two datasets. Different datasets may make the model have different performance.As can be seen in Tables 3 and 4, the model performs better on the U.S. stock forecast. In Table 5, the model performance is relatively poor at HSI prediction.

**Table 3:** Comparison of evaluation indicators of four models on the S&P 500 dataset.

	MSE	MAE	RMSE	R^2^
**LSTM**	0.1208	0.2676	0.3475	0.8829
**WLSTM**	0.1067	0.2470	0.3267	0.8965
**GRU**	0.1000	0.2394	0.3162	0.9030
**WLSTM+Attention**	0.0546	0.1935	0.2337	0.9470

**Table 4:** Comparison of evaluation indicators of four models on the DJIA dataset.

	MSE	MAE	RMSE	R^2^
**LSTM**	0.0785	0.2360	0.2802	0.9235
**WLSTM**	0.0488	0.1646	0.2209	0.9524
**GRU**	0.0452	0.1737	0.2126	0.9560
**WLSTM+Attention**	0.0388	0.1569	0.1971	0.9621

**Table 5:** Comparison of evaluation indicators of four models on the HSI dataset.

	MSE	MAE	RMSE	R^2^
**LSTM**	0.1839	0.3031	0.4288	0.8097
**WLSTM**	0.1335	0.2667	0.3654	0.8618
**GRU**	0.1409	0.2659	0.3754	0.8541
**WLSTM+Attention**	0.1176	0.2433	0.3429	0.8783

**Fig 7:**
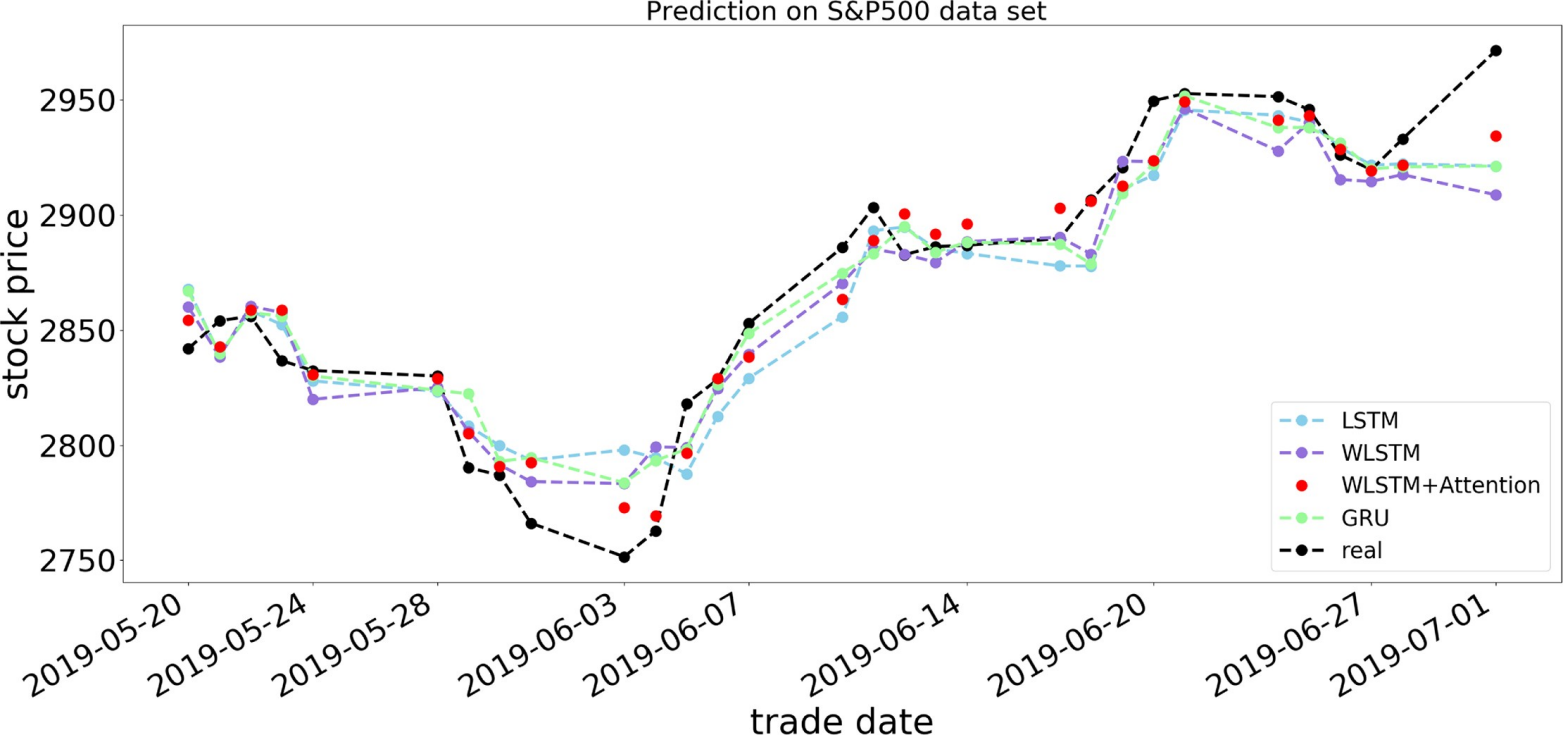
Forecast results of four models for S&P 500 opening price.

**Fig 8:**
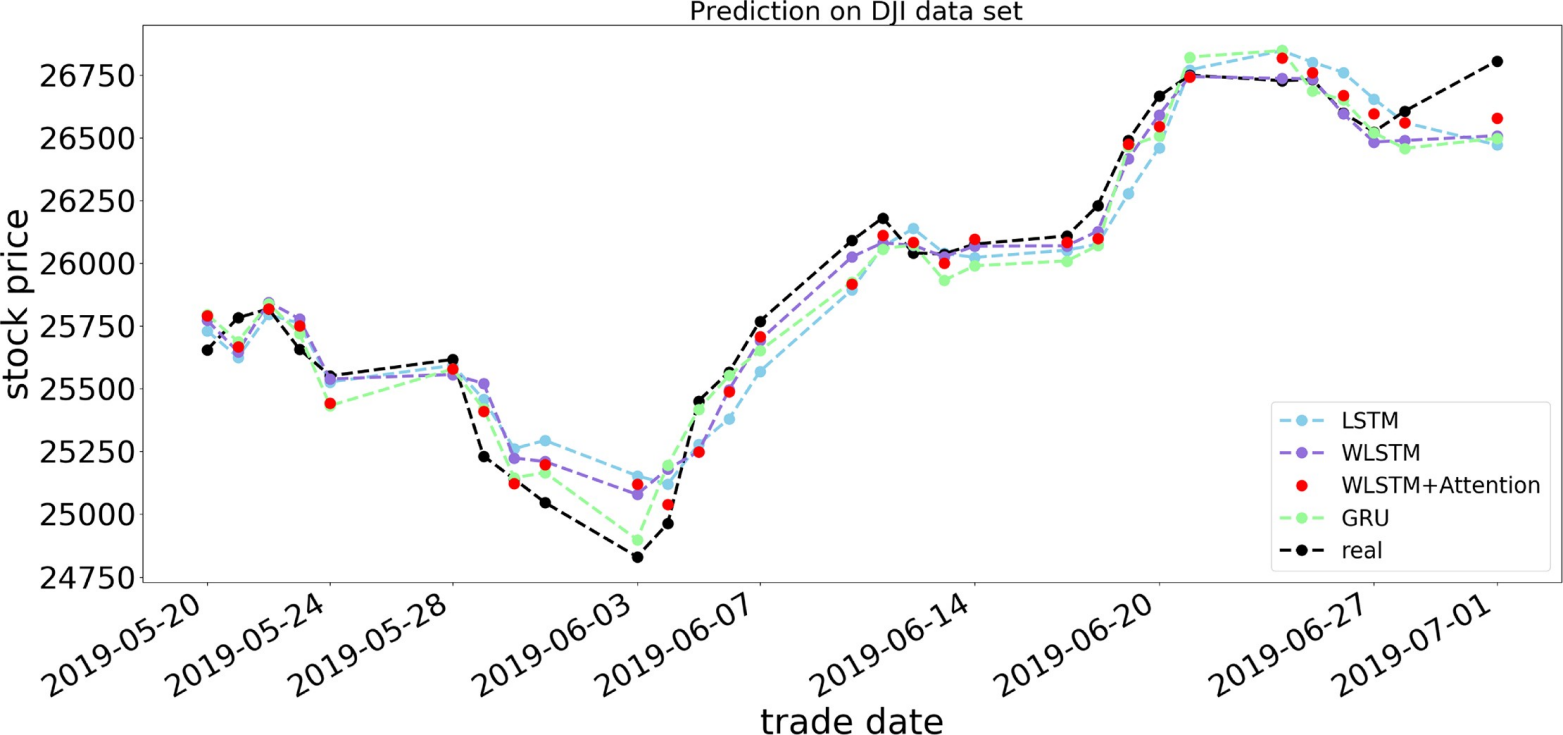
Forecast results of four models for DJIA opening price.

**Fig 9:**
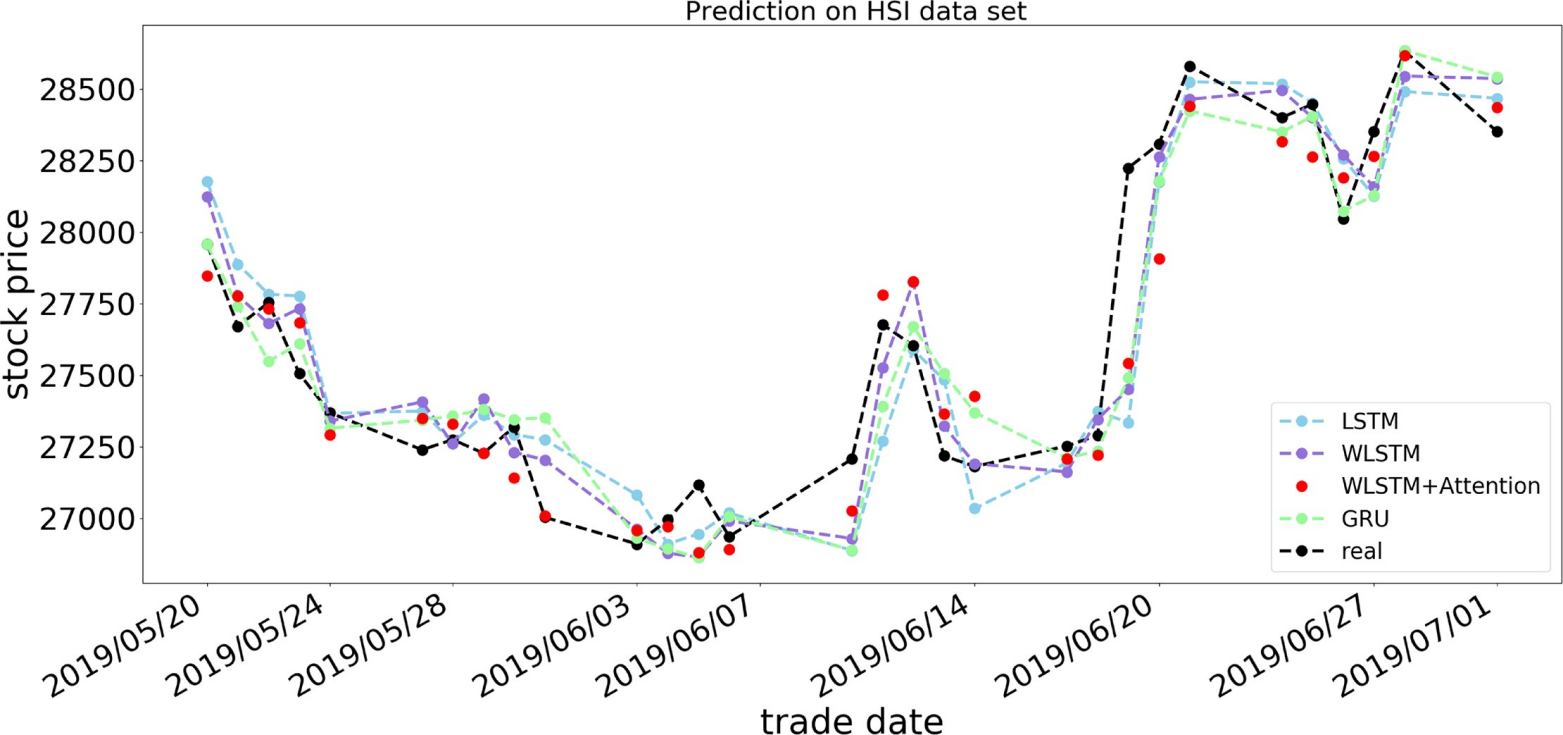
Forecast results of four models for HSI opening price.

Figs 7−9 show the respective opening price prediction results of the four prediction models on the S&P 500, DJIA, and HSI datasets. In the figures, the abscissa is the date corresponding to the stock price, and the ordinate is the opening price of the stock. The blue, purple, red, and green lines, respectively, represent the LSTM model, the LSTM model using only a wavelet transform, the proposed model, and the GRU neural network prediction model. The black line indicates the actual opening price of the current date. From Figs 7 and 8, we can see that the prediction of the opening price of our proposed model on the S&P 500 and DJIA datasets is basically the same as the true value in the trend, and the predicted value basically floats around the true value. When predicting the HSI dataset, it can be seen that the model prediction is not sensitive when many small price fluctuations occur. As shown in Fig 9, from May 24, 2019, to June 3, 2019, the price frequently rises and falls, and the accuracy of the forecast trend at this time decreases, although there are significant differences in the accuracy of the predictions on different datasets. In terms of prediction error, taking RSME as an example, it can be observed from Tables 3−5 that the RSMEs of the proposed model prediction results are 0.2337, 0.1971, and 0.3429, respectively, which are lower than those of other models, indicating the effectiveness of the WLSM+Attention model.

## Conclusion

This paper establishes a forecasting framework to predict the opening prices of stocks. We processed stock data through a wavelet transform and used an attention-based LSTM neural network to predict the stock opening price, with excellent results. The experimental results show that compared to the widely used LSTM, GRU, and LSTM neural network models with wavelet transform, our proposed model has a better fitting degree and improved accuracy of the prediction results. Therefore, the model has broad application prospects and is highly competitive with existing models.

Our future work has several directions. Our work has found that an attention-based LSTM has more predictive outcomes for price prediction than other methods. However, simply considering the impact of historical data on price trends is too singular and may not be able to fully and accurately forecast the price on a given day. Therefore, we can add data predictions related to stock-related news and basic information, so as to enhance the stability and accuracy of the model in the case of a major event.

## Supporting information

S1 File Dataset. (ZIP)
